# Who benefits most from expectancy effects? A combined neuroimaging and antidepressant trial in depressed older adults

**DOI:** 10.1038/s41398-021-01606-1

**Published:** 2021-09-15

**Authors:** Sigal Zilcha-Mano, Meredith L. Wallace, Patrick J. Brown, Joel Sneed, Steven P. Roose, Bret R. Rutherford

**Affiliations:** 1 Department of Psychology, University of Haifa, Mount Carmel, 31905 Haifa, Israel; 2 University of Pittsburgh Department of Psychiatry, Statistics, and Biostatistics, Pittsburgh, PA USA; 3 Columbia University Vagelos College of Physicians and Surgeons, New York State Psychiatric Institute, New York, NY USA; 4 Queens College of the City University of New York, New York, NY USA

**Keywords:** Depression, Prognostic markers

## Abstract

Depressed patients’ expectations of improvement drive placebo effects in antidepressant clinical trials, yet there is considerable heterogeneity in the magnitude of expectancy effects. The present study seeks to identify those individuals who benefit most from expectancy effects using baseline neuroimaging and cognitive measures. Older adult outpatients diagnosed with major depressive disorder (MDD) participated in a prospective, 8-week clinical trial in which expectancy was experimentally manipulated and its effects on depression outcome measured. Based on the literature, we selected a priori 12 cognitive and brain-based variables linked to depression and expectancy, together with demographic variables, and incorporated them into a combined moderator. The combined moderator was developed as a weighted combination of the individual moderators, and was used to identify individuals who benefited most from expectancy effects. The combined moderator was found to predict differential change in depression severity scores between the high- vs. low-expectancy groups with a medium-size effect (Spearman effect size: 0.28). While at the sample level no expectancy effect was found, the combined moderator divided older adults with MDD into those who did and those who did not improve as the result of expectancy manipulation, with those benefiting from the manipulation showing greater processing speed, executive function, and frontostriatal white matter tract integrity. The findings suggest that it is possible to identify a subgroup of older adult individuals with MDD for whom expectancy manipulation results in greater antidepressant treatment response, supporting a precision medicine approach. This subgroup is characterized by distinct cognitive dysfunction and neuroimaging impairments profiles.

## Introduction

Placebo effects are among the most impactful and consistent phenomena in medicine, and they are particularly prominent in major depressive disorder (MDD), the leading cause of disability worldwide [1]. The increase in trial failures in the past decades, which makes it increasingly difficult and expensive to develop effective drugs, is attributed largely to the rising placebo response rather than to declining antidepressant medication (ADM) response [2]. It is imperative to develop methods of minimizing placebo response in antidepressant randomized control trails (RCT) to allow the valid evaluation of new ADMs [2]. At the same time, harnessing the placebo effect in clinical practice can benefit patients. Better mechanistic understanding of placebo effects, including the characteristics of individuals who most benefit from them, has the potential to facilitate achievement of these two important complementary goals.

Patient expectancy, an individuals’ belief about whether and how much they will improve as a result of treatment [3], is a key mechanism underlying the placebo effect [2, 4, 5]. Meta-analyses suggest that expectancy has a large effect in ADM trials, as manifested in smaller ADM effects in placebo-controlled than in open or active comparator trials [6–8], especially as the probability of receiving placebo vs. ADM increases [9, 10]. Our group has developed a methodology to experimentally manipulate expectancy effects prospectively, by randomizing individuals to a high-expectancy group (open trial with a 100% chance of receiving ADM) vs. a low-expectancy group (placebo-controlled trial, where the chances of receiving ADM are lower) [2]. Using this approach, we have reported that it is feasible to manipulate expectancy in young adults and that depressed individuals randomized to high expectancy conditions experience more symptomatic improvement [4]. Findings also demonstrate that in young adults, gains in expectancy during treatment result in subsequent symptom reductions [11].

Expectancy effects may be weaker or more variable in older adults with MDD, as our past studies failed to successfully manipulate expectancy based on the probability of receiving active medication in an antidepressant trial [4, 12]. Older adults with MDD are a population of great interest in identifying moderators of expectancy-based placebo effects because by virtue of brain aging they exhibit variability in cognitive (e.g., memory and executive function) and neural (e.g., integrity of frontostriatal tracts, white matter hyperintensities) markers that may be highly relevant to expectancy [13, 14]. Consistent with this possibility, we recently found that decreased processing speed interfered with expectancy effects in older adults with MDD. There was a trend in the data suggesting that other neurocognitive features may be relevant moderators of expectancy, but the effect sizes for each were small and the individual moderation effects were non-significant [12].

To address this common situation in many RCTs where a set of important moderators is identified, but each one is too small to explain the heterogeneity between individuals, a combined moderator approach has recently been proposed [15–17]. By combining multiple weak moderators into a single stronger moderator of the expectancy effect, a clinically useful index can emerge. Indeed, most studies have failed to distinguish those who do from those who do not benefit from expectancy and placebo effects [18–20]. These generally focus on single clinical and demographical characteristics: short episode duration, few previous episodes, good response to antidepressant treatment, low overall symptom severity [20], gender [19], age [21], and education [22], rather than combinations of variables or brain-based measures. A combined moderator could amplify the effects of weaker, individual moderators. Moreover, each individual moderate alone may provide conflicting treatment indications for a given individual. For example, if individuals with lower education benefit from high expectancy while individuals with high symptom severity show less benefit from expectancy effect, there is no practical guidance for an individual with both lower education and higher symptom severity. Our group has recently demonstrated the benefits of combining different moderators for the purpose of identifying older adults with MDD who may respond to placebo [23].

In the present study, we quantified cognitive and brain-based variables related to expectancy effects and depression to identify a subpopulation of older adults with MDD who benefit most from experimental manipulations of expectancy. We analyzed data from an RCT in which expectancy was experimentally manipulated by randomizing individuals to low- vs. high-expectancy conditions. Twelve potential moderators were chosen a priori based on the literature [24] focusing on cognitive and neuroimaging variables to create a combined moderator of expectancy effect. Specifically, we focused on cognitive performance deficits and white matter hyperintensities on T2-weighted magnetic resonance imaging because they are common in late-life depression, are associated with poor outcomes [13, 14, 25], and have been hypothesized to serve as the mechanisms underlying poorer expectancy effect [12]. We also included education [23] and age [4, 22] because of previous research showing that these variables are associated with placebo effect. Prior to the expectancy manipulation in this RCT, we administered a battery of neuropsychological tests focusing on executive function, complemented by structural MRI and diffusion tensor imaging (DTI), which served as potential moderators.

## Methods

### Participants

The study was conducted at the Clinic for Aging, Anxiety, and Mood Disorders at the New York State Psychiatric Institute (NYSPI). All procedures were approved by the NYSPI Institutional Review Board, and registered on Clinicaltrials.gov (NCT01931202). Eligible participants were men and women aged 60–90 years old, who met Diagnostic and Statistical Manual IV (DSMIV) [26] criteria for non-psychotic MDD, had a 24-item Hamilton Rating Scale for Depression (HRSD) [27] score ≥ 16, were right-handed, gave informed consent, and complied with study procedures.

### Clinical trial design

**Fig. 1:**
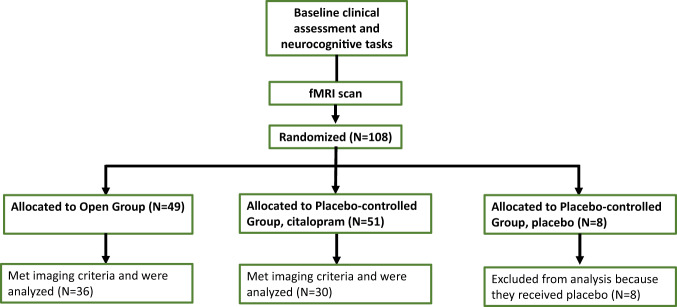
Diagram flow. Diagram flow of individuals participating in the randomized controlled trial. *N* refers to the number of individuals.

Study procedures are described in a previous report [12]. Briefly, 108 patients were enrolled in an 8-week antidepressant clinical trial experimentally manipulating expectancy (Fig. 1). At baseline, patients underwent an initial evaluation to assess eligibility, had pre-randomization psychiatric symptoms and neurocognitive performance measured, and underwent MRI scanning. Next, participants’ expectancy of improvement was experimentally manipulated by randomization to open administration of ADM (i.e., 100% probability of active medication, high expectancy condition) or placebo-controlled administration of ADM (i.e., perceived 50% probability of active medication, low-expectancy condition). This experimental procedure has been successful in manipulating the expectancy effect in multiple previous studies [4, 12]. Participants receiving open medication began either escitalopram or duloxetine (depending on their past treatment history), while those in the placebo-controlled condition were randomized to medication or placebo in a 6:1 ratio favoring medication. Clinicians and participants were aware of group assignment (i.e., open vs. placebo-controlled) but blinded to treatment assignment within the placebo-controlled group, whereas outcome assessors were blinded to both group and specific treatment assignment.

### Measures

Neurocognitive tasks. (a) Stroop Color-Word Test, measuring response inhibition, adjusted for age and education [28], (b) Digit Symbol subtest from the WAIS-III [29], and (c) Initiation/Perseveration (I/P) subtest of the Mattis Dementia Rating Scale (DRS) [30].

Neuroimaging procedures. A GE Discovery MR750 3.0 Tesla whole-body scanner (GE Medical Systems, Waukesha, Wisconsin) and 48-channel head coil were used. A 3-plane localizer (scout) was used to determine patient position, followed by T1-weighted (FSPGR), T2 fluid-attenuated inversion recovery (FLAIR), and DTI scans. T2 FLAIR scans quantified whole-brain WMH volume in cm. DTI data were processed using FMRIB Software Library (FSL) version 6.0.1 (Oxford, UK) and analyzed with tract-based spatial statistics (TBSS). These methods provided mean fractional anisotropy (FA) and mean diffusivity (MD) values for the superior longitudinal fasciculus (SLF) and uncinate fasciculus (UNC) for each individual. Detailed descriptions of neuroimaging procedures appear in the Supplementary Materials.

### Statistical analyses

We adopted a combined moderator approach to address the problem of weak individual moderator effects in clinical trials research [16, 17]. All moderators were standardized. We first created a new data set from all possible pairs of a patient in the high-expectancy and a patient in the low-expectancy conditions (*n*_1_*n_2_ pairs, where *n*_i_ is the number of patients in condition *I*, *i* = 1,2). For each pair, we calculated the average of their moderator values (for each moderator), and the difference in their estimated HRSD slopes. The slope of each patient was estimated using a mixed-effect regression model, with random slope and intercept for each patient, based on a linear trend in log of week. For each potential moderator, we computed the non-parametric Spearman correlations in the new data between the differences in HRSD slopes and the covariate average across all pairs. Non-parametric Spearman correlation was used to allow for non-normal moderations and to reduce the potential influence of outliers in the data.

Second, we created the combined moderator, which is an optimally weighted combination of individual moderators. The weight assigned to each moderator was estimated using a LASSO regression with the glmnet package in R. The dependent variable in the Lasso regression was the slope difference of each pair, and the predictors were the averages of the potential moderators. The Lasso regression is a linear regression, but it uses a penalty on coefficient absolute values, which shrinks their estimate. The purpose of the shrinkage is to avoid overfitting. Unimportant predictors are expected to shrink to zero. The estimated coefficients (standardized to sum to 1 in absolute values) are used as weights for calculating the combined moderator score of each individual. In this way, the combined moderator represents an optimally weighted linear combination of the individual moderator scores. The resulting effect is measured by the correlation of the averages of the combined moderator (M) and the paired slope differences (in the paired data), denoted by Cor(M). We used bootstrapping to obtain a confidence interval. We resampled with replacement patients, separately from each condition, and applied all the procedures above, resulting in 1000 estimates of the correlation. The 2.5 and 97.5 quantiles of the estimates served as confidence intervals. Finally, we assessed the discriminative utility of the combined moderator, using a linear regression of expectancy condition, the combined moderator, and the combined moderator by condition interaction on treatment outcome.

## Results

### Demographic and clinical characteristics

Of the 108 patients participating in the RCT, 8 received placebo and therefore were excluded from the analyses. A total of 66 individuals who received ADM underwent baseline MRI scanning, creating the effective sample for this analysis. Of these patients, 36 were randomized to the high expectancy and 30 to the low-expectancy group (Fig. 1). No significant differences in demographic data, baseline clinical characteristics or outcome were found between participants who were and were not scanned (Table S1 in the online supplements) or between participants in the Placebo-controlled and Open groups (Table S2 in the online supplements). The two groups did not differ significantly also in pre-treatment depression scores (23.7 vs. 22.5 in the Placebo-controlled vs. Open group, respectively; p = .38). The two groups did not differ significantly in their HRSD slopes from pre-treatment to post treatment (–0.27 vs. 0.76 in the Placebo-controlled vs. Open group, respectively; *p* = 0.096), suggesting the need to identify potential moderators to explain the variability in this effect.

### Creating the combined moderator

Across the entire dataset, there were only 15 missing observations (6 in WMH and 9 in education years) in the 12-variables of interest. The few missing observations were imputed using MissForest. As expected, the effect for the individual moderators was small and included both positive and negative correlations (Table 1). Positive correlations mean that higher levels of the moderator were associated with less improvement in treatment outcome in the high vs. low-expectancy condition. The largest individual moderators were fractional anisotropy (FA) values in the anterior thalamic radiations (left and right), followed by left superior longitudinal fasciculus FA and Stroop Color-Word score, all showing a positive correlation. The largest negative correlations were found for right uncinate fasciculus FA and Mattis DRS Initiation/Perseveration subscale score.

**Table 1:** Individual moderator effect sizes and their weights in the combined moderator.

	Correlation	Weight
WAIS digit symbol	−0.01	−0.09
Stroop	0.14	0.16
WMH	−0.05	0.00
Mattis DRS initiation/perseveration	−0.06	−0.01
DTI: Anterior thalamic radiation L	0.16	0.03
DTI: Anterior thalamic radiation R	0.16	0.13
DTI: Superior longitudinal fasciculus L	0.14	0.19
DTI: Superior longitudinal fasciculus R	0.07	−0.10
DTI: Uncinate fasciculus L	0.01	−0.15
DTI: Uncinate fasciculus R	−0.08	−0.05
Age	−0.03	−0.04
Education	−0.02	0.06
Combined moderator	0.28	

### The combined moderator effect

**Fig. 2:**
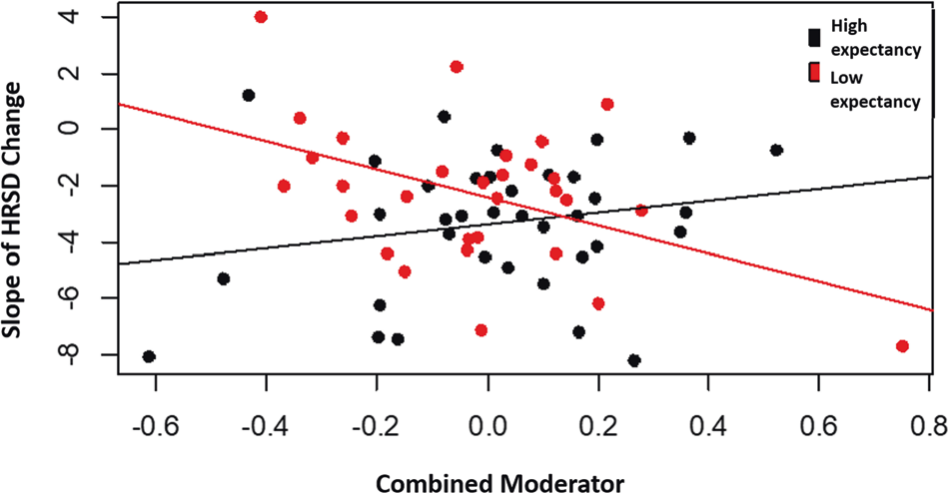
Combined moderator by expectancy interaction. The linear interaction of the combined moderator and expectancy condition in predicting the slope of symptom reduction.

The combined moderator had a larger effect size than any individual moderator (effect size = 0.28 (95% CI: .27, .67 vs. .01 ≤ *R* ≤ 0.16 for the individual moderators). The linear interaction of the combined moderator and expectancy condition is shown in Fig. 2. When the combined moderator is lower than the cross-point (0.14), the high-expectancy condition showed more symptom reduction than the low-expectancy condition (Cohen’s *d* = 0.58; −7.41 vs. −4.46 points reduction in HRSD for the high- vs. low-expectancy conditions). When the combined moderator is higher than the cross-point, the differences were not substantial (Cohen’s *d* = 0.12; −7.17 vs. −8.03 points reduction in HRSD for the high- vs. low-expectancy conditions). The effect sizes after removing each moderator separately appear in Table S3 of the online supplements. As a post hoc analysis, we repeated this procedure adding sex, ethnicity, and race. Findings appear in Table S4 of the online supplements.

## Discussion

The present findings reveal that some depressed older adults benefited more than others from the experimental manipulation of expectancy during antidepressant treatment. Specifically, participants with a lower combined moderator score experienced significantly greater reductions in HRSD scores in the high- vs. low-expectancy conditions compared to other participants who benefited substantially less from the expectancy manipulation. These two distinct subgroups were concealed when focusing on each potential moderator separately: only small effects ranging in size between 0.01 ≤ *R* ≤ 0.16 were observed for each separate neurocognitive moderator. The combined effect was much larger (*R* = 0.28), and was able to differentiate between those who benefit from expectancy manipulation (with an effect size of Cohen’s *d* = 0.58) and those who do not (Cohen’s *d* = 0.12).

Contingent upon future external validation and prospective research, the combined moderator identified in the present study can be used as a clinically useful index to predict who is likely to benefit from an intervention to enhance expectancy. That is, upon assessing a new patient and having access to the measurements contained in the combined moderator, these values could be used to obtain an estimate of the patient’s likelihood of benefiting from a high expectancy compared to a lower expectancy intervention. This may have implications for future randomized trial inclusion criteria as well as for daily clinical practice. The index may be used to direct efforts for developing new drugs for the populations that are less expected to show an expectancy effect, which may facilitate drug/placebo signal detection by minimizing placebo response. That is, this type of analysis may be useful in predicting a randomized controlled trial participant’s likelihood of demonstrating a high placebo response. Such information may be useful in designing selection criteria and/or stratifying samples so as to maximize signal detection for novel therapeutic agents. At the same time, the use of the combined moderator may allow targeting of patients who are likely to benefit from expectancy effects with enhanced psychoeducation about the potential benefits of treatment. Based on the index, the attending physician may be able to use expectancy augmentations techniques in combination with the ADM, for example, by further informing the patient about the expected positive effects of a given drug.

The present findings can also shed light on the critical capabilities required for showing an expectancy effect, answering questions that have been of great interest in the empirical literature on placebo effect. The findings may suggest that of the capabilities evaluated here, executive functioning and frontostriatal tract integrity, are especially critical. Intact executive functioning may be critical in reappraising the responses to an event according to new information presented in the world (in this case, verbal information regarding the probability of receiving an active drug). Such new information is then processed and evaluated through circuits implicated in generating an expectancy effect. Reduced integrity of the frontostriatal tract may interrupt the modulation of limbic and striatal structures necessary to reduce depressive symptoms as a result of the expectancy manipulation. This potential mechanistic explanation for the expectancy effect is consistent with previous findings demonstrating the importance of executive functioning in producing expectancy effects and in symptom reduction in older adults with MDD [25]. It is also consistent with previous findings suggesting that DTI may be implicated in ADM non-response in late life depression [12].

The approach used in the present study goes beyond previous research, by combining distinct moderators to better capture the richness and complexity of the neurocognitive capacities that are needed to benefit from the expectancy effect. It has the potential to leverage current research on placebo responders by combining the weak moderators identified so far in a way that captures their specific importance. The present findings are consistent with previous ones demonstrating the promise of this approach in translating heterogeneity in clinical outcomes into personalized recommendations [31–33]. To be validated, the algorithm must be prospectively tested. One potential test is by randomizing individuals to either (a) receive randomly high- vs. low-expectancy manipulation, or (b) be assigned to high- vs. low-expectancy manipulation using the algorithm specified in the current study (namely, stratifying individuals by whether they are above or below the cut-point of the combine moderator). We expect the assignment by algorithm to vastly outperform the random assignment. Future research should further test how weights could be adjusted to tailor the algorithm to diverse populations.

The most important limitation of the current study is that our sample size was restricted by the unique characteristics of the sample that underwent the experimental manipulation of expectancy and required each patient to have rich and detailed cognitive and neuroimaging characteristics. Future studies should use a larger sample and test the validity of the prediction on an external sample. Until then, the findings should be regarded as exploratory. Notwithstanding these limitations, the findings identify a subpopulation of older adults with MDD who benefitted from expectancy manipulation: those individuals with intact executive functioning (enabling reappraising responses based on the new expectancy-related information arriving), as well as less reduced integrity of the frontostriatal tract (enabling the modulation of limbic and striatal structures). These findings have the potential to greatly advance our understanding of the pathogenesis of late-life depression, and shed light on the biology of the expectancy effect in antidepressant response. The findings further hold the potential for improving the efficacy of treatment of late life depression through more precise treatment selection, focusing on psychoeducational interventions [34] and on interventions aimed at improving response inhibition [35].

## Supplementary information

Online Supplements
