# Correction to: Who benefits most from expectancy effects? A combined neuroimaging and antidepressant trial in depressed older adults

**DOI:** 10.1038/s41398-022-02169-5

**Published:** 2022-10-04

**Authors:** Sigal Zilcha-Mano, Meredith L. Wallace, Patrick J. Brown, Joel Sneed, Steven P. Roose, Bret R. Rutherford

**Affiliations:** 1 Department of Psychology, University of Haifa, Mount Carmel, 31905 Haifa, Israel; 2 University of Pittsburgh Department of Psychiatry, Statistics, and Biostatistics, Pittsburgh, PA USA; 3 Columbia University Vagelos College of Physicians and Surgeons, New York State Psychiatric Institute, New York, NY USA; 4 Queens College of the City University of New York, New York, NY USA

**Keywords:** Depression, Prognostic markers

Correction to: *Translational Psychiatry* 10.1038/s41398-021-01606-1, published online 15 September 2021

The original version of this article unfortunately contained an error in the references. Reference 12 should read as follows: Rutherford BR, Choi CJ, Choi J, Maas B, He X, O’Boyle K, et al. Slowed processing speed disrupts patient expectancy in late life depression. Am J Geriatr Psychiatry. 2021;29:619–30. The original article has been corrected.

