# Supplementary material for: Who benefits most from expectancy effects? A combined neuroimaging and antidepressant trial in depressed older adults
Source: Transl Psychiatry. 2021 Sep 15;11:475. doi: 10.1038/s41398-021-01606-1 (PMC8443672; doi:10.1038/s41398-021-01606-1)
Supplement: Supplementary file 1 — Online Supplements [file 41398_2021_1606_MOESM1_ESM.docx]

Diffusion tensor imaging (DTI) Data Acquisition

DTI enables the mapping of white matter hyperintensity (WM) tracts through the measurement of the diffusion of water molecules, which can either be isotropic and diffuse equally in all directions in the absence of barriers, or be anisotropic and diffuse along the axis of existing barriers^28,29^. WM tracts in the brain form organized boundaries along which water will diffuse thus enabling anisotropic diffusion, which is often indexed via fractional anisotropy (FA). An intact boundary will direct diffusion along one axis, resulting in a higher FA value, while a damaged tract will result in less anisotropic diffusion, resulting in a lower FA value^[[1]](#endnote-1)^. In addition to FA, mean diffusivity (MD), radial diffusivity (RD), and axial diffusivity (AD) can all be computed using DTI. Generally, FA is considered a primary and overall measure of WM integrity, while RD and AD are sensitive to demyelination and axonal degeneration, respectively^[[2]](#endnote-2)^. MD is thought to be sensitive to cellularity, edema and necrosis^[[3]](#endnote-3)^. Therefore, we used DTI to conduct analysis on white matter integrity and its role in cognitive function as well as treatment outcome.

DTI data were acquired on a GE Discovery MR750 3.0 Tesla whole body scanner (GE Medical Systems, Waukesha, Wisconsin) using a spin-echo echo planar imaging (SE-EPI) sequence with the following parameters: 30 non-collinear spatial directions at b value = 1000 s/mm^2^, three baseline images at b = 0 s/mm^2^ , TR/TE = 9500 ms /86.6 ms, flip angle = 90 degrees, field of view = 24 cm, matrix size = 132 × 128 (machine-interpolated to 256 × 256 for post-processing, in-plane resolution of 0.94 mm), 60 axial slices, slice thickness = 2 mm without gap, number of excitations (NEX) = 2, scan time for each excitation was 5 minutes 13.5 seconds and we averaged 2 excitations for each subject.

DTI Data Preprocessing and Analysis

DTI data were processed using FMRIB Software Library (FSL) version 6.0.1 (Oxford, UK)^[[4]](#endnote-4)^. Specifically, DTI data were corrected for subject movement, eddy current-induced distortion, outlier replacement, and within-volume (or "slice-to-volume") movement using FSL Edd^[[5]](#endnote-5),^^[[6]](#endnote-6)^. Brain Extraction Tool^[[7]](#endnote-7)^ was used to extract a brain mask from the eddy corrected image in order to exclude skull and non-brain tissue. Diffusion tensor was fitted using FSL DTIFIT for each voxel and the fitted diffusion tensors were used to generate the FA and color encoded FA images. Data quality was assessed by visually inspecting the eddy corrected diffusion images and the color encoded FA images^[[8]](#endnote-8)^ to exclude subjects who had motion-corrupted or signal loss DTI data. We also visually checked the extracted brain masks to make sure that skull and non-brain tissue were completely removed.

We then ran TBSS^10^ on the FA images for those subjects who passed the quality control. All FA images were aligned to a 1x1x1mm standard space using nonlinear registration on the adult-derived target image FMRIB58_FA provided by FSL. A mean FA image was created by averaging the aligned FA images across all subjects and was skeletonized to generate a mean FA skeleton. The FA threshold for the skeletonization was 0.20 to exclude gray matter regions from the analyses. We then project all subjects’ FA images onto the mean FA skeleton which was then used for statistical analyses. Nonlinear warps and skeleton projection were then also applied to MD, AD, and RD images for statistical analyses.

**Table S1. Baseline characteristics of those scanned vs. not scanned.**

|  | **Scanned** **(n=66)** | **Not scanned** **(n=34)** | **Between-Group Difference** |
| --- | --- | --- | --- |
| **Characteristic** | **n/mean(SD)** | **n/mean(SD)** | **p-value** |
| Condition  Open trial  Placebo-controlled | 36  30 | 13  21 | .14 |
| Age | 69.1(SD=6.8) | 71.6(SD=9.3) | .16 |
| Sex  Male  Female | 23  43 | 13  21 | .83 |
| Race  Asian  Black  White  >1  Do not know | 1  8  49  3  5 | 0  6  20  6  1 | .65 |
| Ethnicity  Not Hispanic/ Latino  Hispanic/Latino | 56  10 | 27  7 | .57 |
| Years of education | 16.7(SD=2.9) | 15.6(SD=2.6) | .09 |
| 24-item Hamilton Rating Scale for Depression | 23.0(SD=5.5) | 23.7(SD=7.4) | .64 |

**Table S2. Baseline characteristics for the present sample by treatment condition.**

|  | **Open trial Group (n=36)** | **Placebo-controlled Group (n=30)** | **Between-Group Difference** |
| --- | --- | --- | --- |
| **Characteristic** | **n/mean(SD)** | **n/mean(SD)** | **p-value** |
| Age | 68.5(SD=6.8) | 69.8(SD=6.9) | .43 |
| Sex  Male  Female | 11  25 | 12  18 | .44 |
| Race  Asian  Black  White  >1  Do not know | 1  6  25  1  3 | 0  2  24  2  2 | .65 |
| Ethnicity  Not Hispanic/ Latino  Hispanic/Latino | 29  7 | 27  3 | .32 |
| Years of education | 17(SD=2.6) | 16.2(SD=3.4) | .17 |
| 24-item Hamilton Rating Scale for Depression | 22.5(SD=5.9) | 23.7(SD=4.9) | .38 |

Table S3. Effect sizes of the combined moderator after removing each moderator separately.

|  | Effect size |
| --- | --- |
| WAIS digit symbol | 0.29 |
| Stroop | 0.27 |
| WMH | 0.32 |
| Mattis DRS Initiation/Perseveration | 0.32 |
| DTI: Anterior thalamic radiation L | 0.32 |
| DTI: Anterior thalamic radiation R | 0.32 |
| DTI: Superior longitudinal fasciculus L | 0.30 |
| DTI: Superior longitudinal fasciculus R | 0.32 |
| DTI: Uncinate fasciculus L | 0.29 |
| DTI: Uncinate fasciculus R | 0.33 |
| Age | 0.32 |
| Education | 0.31 |
| Sex | 0.32 |
| Ethnicity | 0.29 |
| Race | 0.32 |

Table S4. Individual moderator effect sizes and their weights in the combined moderator, including sex, ethnicity and race.

|  | Correlation | Weight |
| --- | --- | --- |
| WAIS digit symbol | -0.01 | -0.11 |
| Stroop | 0.14 | 0.16 |
| WMH | -0.05 | -0.01 |
| Mattis DRS Initiation/Perseveration | -0.06 | -0.02 |
| DTI: Anterior thalamic radiation L | 0.16 | 0.02 |
| DTI: Anterior thalamic radiation R | 0.16 | 0.11 |
| DTI: Superior longitudinal fasciculus L | 0.14 | 0.14 |
| DTI: Superior longitudinal fasciculus R | 0.07 | -0.06 |
| DTI: Uncinate fasciculus L | 0.01 | -0.10 |
| DTI: Uncinate fasciculus R | -0.08 | -0.03 |
| Age | -0.03 | -0.04 |
| Education | -0.01 | 0.04 |
| Sex | -0.02 | -0.01 |
| Ethnicity | -0.16 | -0.13 |
| Race | 0.09 | -0.02 |
| Combined moderator | 0.32 |  |

1. **References**

   Shenton ME, Hamoda HM, Schneiderman JS, et al. A review of magnetic resonance imaging and diffusion tensor imaging findings in mild traumatic brain injury. *Brain Imaging Behav*. 2012;6(2):137-192. doi:10.1007/s11682-012-9156-5 [↑](#endnote-ref-1)
2. Alexander AL, Lee JE, Lazar M, Field AS. Diffusion tensor imaging of the brain. *Neurother J Am Soc Exp Neurother*. 2007;4(3):316-329. doi:10.1016/j.nurt.2007.05.011 [↑](#endnote-ref-2)
3. Alexander AL, Hurley SA, Samsonov AA, et al. Characterization of cerebral white matter properties using quantitative magnetic resonance imaging stains. Brain Connect. 2011;1(6):423-446. doi:10.1089/brain.2011.0071 [↑](#endnote-ref-3)
4. Smith SM, Jenkinson M, Woolrich MW, et al. Advances in functional and structural MR image analysis and implementation as FSL. In: NeuroImage. ; 2004. doi:10.1016/j.neuroimage.2004.07.051 [↑](#endnote-ref-4)
5. Andersson JLR, Graham MS, Drobnjak I, Zhang H, Filippini N, Bastiani M. Towards a comprehensive framework for movement and distortion correction of diffusion MR images: Within volume movement. Neuroimage. 2017;152:450-466. doi:10.1016/j.neuroimage.2017.02.085 [↑](#endnote-ref-5)
6. Andersson JLR, Graham MS, Zsoldos E, Sotiropoulos SN. Incorporating outlier detection and replacement into a non-parametric framework for movement and distortion correction of diffusion MR images. Neuroimage. 2016;141(1 November 2016):556-572. doi:10.1016/j.neuroimage.2016.06.058 [↑](#endnote-ref-6)
7. Smith SM. Fast robust automated brain extraction. Hum Brain Mapp. 2002;17(3):143-155. doi:10.1002/hbm.10062 [↑](#endnote-ref-7)
8. He X, Liu W, Li X, et al. Automated assessment of the quality of diffusion tensor imaging data using color cast of color-encoded fractional anisotropy images. Magn Reson Imaging. 2014;32(5):446-456. doi:10.1016/j.mri.2014.01.013 [↑](#endnote-ref-8)
